# GNNMutation: a heterogeneous graph-based framework for cancer detection

**DOI:** 10.1186/s12859-025-06133-0

**Published:** 2025-06-04

**Authors:** Nuriye Özlem Özcan Şimşek, Arzucan Özgür, Fikret Gürgen

**Affiliations:** Department of Computer Engineering, Boğaziçi University, Bebek, İstanbul, 34342 Turkey

**Keywords:** Dna mutations, Protein-protein interactions, Graph neural networks, Explainer, Cancer detection, Causal genes

## Abstract

**Background:**

When genes are translated into proteins, mutations in the gene sequence can lead to changes in protein structure and function as well as in the interactions between proteins. These changes can disrupt cell function and contribute to the development of tumors. In this study, we introduce a novel approach based on graph neural networks that jointly considers genetic mutations and protein interactions for cancer prediction. We use DNA mutations in whole exome sequencing data and construct a heterogeneous graph in which patients and proteins are represented as nodes and protein-protein interactions as edges. Furthermore, patient nodes are connected to protein nodes based on mutations in the patient’s DNA. Each patient node is represented by a feature vector derived from the mutations in specific genes. The feature values are calculated using a weighting scheme inspired by information retrieval, where whole genomes are treated as documents and mutations as words within these documents. The weighting of each gene, determined by its mutations, reflects its contribution to disease development. The patient nodes are updated by both mutations and protein interactions within our noval heterogeneous graph structure. Since the effects of each mutation on disease development are different, we processed the input graph with attention-based graph neural networks.

**Results:**

We compiled a dataset from the UKBiobank consisting of patients with a cancer diagnosis as the case group and those without a cancer diagnosis as the control group. We evaluated our approach for the four most common cancer types, which are breast, prostate, lung and colon cancer, and showed that the proposed framework effectively discriminates between case and control groups.

**Conclusions:**

The results indicate that our proposed graph structure and node updating strategy improve cancer classification performance. Additionally, we extended our system with an explainer that identifies a list of causal genes which are effective in the model’s cancer diagnosis predictions. Notably, some of these genes have already been studied in cancer research, demonstrating the system’s ability to recognize causal genes for the selected cancer types and make predictions based on them.

## Background

Cancer is one of the leading causes of death globally [1], with its development driven by genetic alterations that disrupt normal cellular processes, leading to uncontrolled growth and metastasis. The genetic changes play a central role in the initiation and progression of tumors [1]. Various types of genetic data have been used for cancer research, such as gene and RNA expression levels [2–12] and DNA methylation patterns [3, 5, 8, 9, 13, 14]. However, the main source of genetic information is DNA and can be accessed through DNA sequencing techniques.

Recent advancements in DNA sequencing technologies have significantly enhanced the speed, accuracy, and cost-effectiveness of sequencing, facilitating the creation of large sequencing databases. In [15, 16], whole genome sequencing data are utilized and processed with various statistical methods and available genomics tools to detect novel biomarkers for cancer. Besides statistical methods, machine learning methods are of great importance to draw meaningful inferences from the vast amount of data generated. Genetic mutations can be identified using the sequencing data by variant calling methods. Mutations can be analyzed directly, rather than working with the raw genomic sequences, in order to reduce the computational load on processing systems. Mutation data, in the form of single nucleotide variants (SNVs) and copy number variations (CNVs), have been used to explore the genetic basis of cancer in recent studies [3, 5, 8, 9, 13, 14]. Mutations in genes can also alter protein function since genes provide the instructions for protein synthesis. Such changes can disrupt protein-protein interactions, potentially impacting cellular processes such as growth and spread and eventually contribute to tumorigenesis [1]. In this study, we propose an approach that integrates information on both mutations and protein interactions for cancer prediction.

Proteins engage in numerous interactions to facilitate a wide array of biological processes [17]. These Protein-Protein Interactions (PPIs) can be represented as a graph, where proteins are the nodes and the interactions between them form the edges. With recent advancements in graph-based neural networks, PPI networks have become an essential tool for genetic disease-related studies, providing a structured representation of the complex relationships between proteins [2]. For example, [3] introduced a graph-based framework for novel cancer gene discovery, leveraging Graph Neural Networks (GNNs) for node-level classification. In this approach, genes within the PPI network were labeled based on their relevance to cancer (either cancer-related or non-cancer-related). Graph Convolutional Networks (GCNs) were then applied to predict whether unlabeled genes were associated with cancer. In another study [4], a PPI network was constructed for each individual patient, and the network was classified using a Graph Isomorphism Network (GIN) architecture to detect disease subnetworks. Another form of graph used in disease-related studies is the patient similarity network. In these networks, patients are represented as nodes, with omics data serving as the features for each node. Edges are formed by calculating a similarity score between the patient features, and an edge is created between two nodes if their similarity score exceeds a predefined threshold. Patient similarity networks have been processed using GNNs in recent cancer studies for tasks such as survival analysis, as well as cancer type and subtype classification [18–20]. These studies primarily utilize homogeneous graphs, where a single type of entity (proteins or patients) is represented as nodes. In PPI-based studies, proteins serve as the nodes, and patient features are mapped onto them. Similarly, in patient similarity networks, patients are the nodes, and protein interactions are not incorporated into the model. However, a more comprehensive approach can involve heterogeneous graphs, where both patients and proteins are represented as distinct types of nodes. This approach would allow for the analysis of the relation between protein interactions and the cancer state of patients through the message passing mechanism of the graph.

In this study, we propose an attention-based graph neural network (GNN) with a heterogeneous graph structure, named GNNMutation, designed to classify cancer patients based on DNA mutations and protein interactions. The gene mutation data from the UK Biobank [21] were used, encompassing a cohort of patients both with and without a cancer diagnosis. Among the various types of cancer, breast cancer is the most prevalent in women, while prostate cancer in men [22]. Lung and colon cancers rank as the second and third most common types in both sexes [22]. Accordingly, we have selected these four cancer types for our case study. In addition to leveraging DNA mutation data, we enrich this information with protein-protein interaction (PPI) data to enhance the model’s predictive capability. GNNMutation operates in a heterogeneous graph environment, integrating patient mutation data with PPI data. The graph structure defines two distinct types of nodes: patients and proteins. Gene mutations and protein interactions are represented as edges within the graph. To the best of our knowledge, this is the first study to conceptualize patients as separate nodes while combining patient data, mutations, and proteins in a unified heterogeneous graph structure. In GNNMutation, we not only incorporate protein-protein interaction edges but also define directed edges from protein nodes to patient nodes, based on corresponding gene mutations. These protein-to-patient edges enable the transfer of information from proteins to patients, enhancing the overall information flow and improving classification performance. GNNMutation demonstrates a high capacity for distinguishing between cancer cases and control groups. The explanatory part of GNNMutation specifies the causal genes that lead to the development of the disease.

The key contributions of our work can be summarized as follows:

1. A novel heterogeneous graph structure has been proposed for the representation of patients, DNA mutations and proteins for disease classification.
2. Both the protein interaction information and mutation information have been transferred into the decision mechanism by defining corresponding edges.
3. The parameters of the classification model have been examined to identify a list of causal genes that reflect the effectiveness of the proposed heterogeneous graph modeling approach.

## Methods

**Fig. 1:**
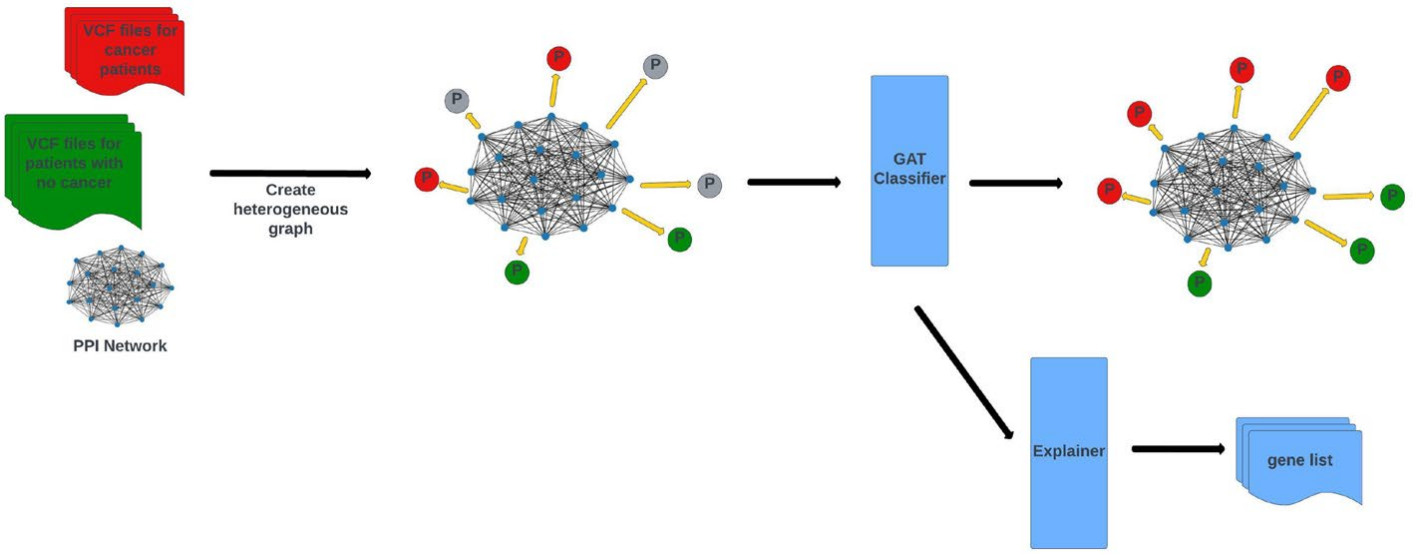
Overview of GNNMutation: The input of the framework is a heterogeneous graph modeling DNA mutations and protein interactions. GAT classifier is applied to decide whether a patient has cancer or not. The explainer module examines classifier parameters and outputs a list of predicted causal genes

In this study, we propose a heterogeneous graph-based framework for cancer detection. The input of the system is a novel heterogeneous graph that models patients, DNA mutations and proteins in a single graph. This graph represents the protein-protein interactions and DNA mutations by defining edges between the corresponding patient and protein nodes. A graph attention network (GAT) classifier is applied on this graph to decide whether an unlabeled patient has cancer or not. An explainer module in the framework examines the GAT model parameters and suggests a list of causal genes for each cancer type. Figure 1 shows an overview of the proposed framework.

### Dataset

We utilized the variant call format (VCF) files [23] of the whole exome sequencing data from UKBiobank [21]. The sequencing data is obtained from blood samples. Therefore, no biopsy or tumor sample is required for our framework. From the data to which we have access rights, we randomly selected 1000 patients diagnosed with the cancer type that we investigate. For the control group, we randomly selected the same number of patients without a cancer diagnosis. Four most common cancer types are tested with the proposed method: breast, prostate, lung and colon.

Our gene set is based on the mutated genes in the VCF files. Then we selected genes that are also listed in the Hallmark gene sets [24]. From this set, we selected genes that produce proteins in the physical protein-protein interaction (PPI) network of StringDB [25]. Based on the resulting list of genes, we selected a subset of the physical PPI network of StringDB and used it as the PPI network for our tests.

### GNNMutation

#### Heterogeneous graph

**Fig. 2:**
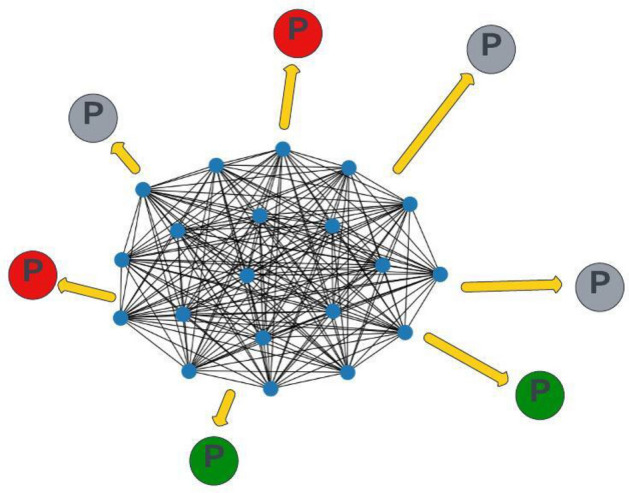
Heterogeneous graph of patients and genes: Small blue nodes are the gene nodes and make a PPI network among themselves. Big nodes with P letter are the patient nodes. Red represents cancer patients, green represents no cancer patients and grey represents test nodes to be predicted

We formulate our problem as a node classification problem on a global graph. We have defined a heterogeneous graph model to represent patients, mutations and proteins together. To the best of our knowledge, this is the first study to construct a heterogeneous graph that combines protein and mutation information and defines patients as separate nodes. We constructed a global graph $G = (P, Pr, E_{ppi}, E_{mut})$ where *P* represents the patient nodes, *Pr* represents the protein nodes, $E_{ppi}$ represents the edges from the PPI network, and $E_{mut}$ represents the edges based on mutations. We used proteins as nodes and defined the edges between proteins based on the StringDB physical PPI network. The protein-protein edges ($E_{ppi}$) in the graph were defined as undirected. We used patients as nodes and defined edges from proteins to patients if there is a mutation in the gene that produces the protein for that patient. The protein-patient edges ($E_{mut}$) in the graph were defined as directed. In this way, the patient nodes are updated directly by mutation information and indirectly by protein interactions. The protein nodes are only updated by the PPI network. Based on these updates, the network predicts the class label for test patients. An example input graph can be found in Fig. 2. In the figure, the PPI network is in the center. The patient nodes are scattered over the PPI. The small blue nodes are the protein nodes (*Pr*). The protein-protein edges ($E_{ppi}$) are represented as black lines. The large nodes are the patient nodes (*P*). The red and green *P* nodes are the train patient nodes and the grey *P* nodes are the test patient nodes to be predicted. Protein-patient edges ($E_{mut}$) are represented as yellow arrows. For patient nodes, we calculated mutation importance vectors for each patient and used them as node features. For protein nodes, we assigned one-hot vectors for each protein and used them as node features. The length of the one-hot vector was equal to the number of proteins in the graph. For each protein, the coordinate of the 1 element in the one-hot feature vector was different. In other words, all proteins had different feature vectors.

#### Patient feature vectors

For each patient, we calculated a feature vector that summarizes the significance of each mutation information in the VCF files. We applied the feature type that performed best in our previous study [26], namely *bm25-tf-rf*. In [26], cancer classification was performed with different feature weighting methods containing *bm25-tf-rf* and other statistical and mutation scoring schemes and the test results suggested that *bm25-tf-rf* outperformed other feature weighting methods. Term-frequency relevance-frequency (*tf-rf*) is a supervised statistical measure originally proposed in the field of text-based information retrieval [27]. We can clarify the meaning of the term *tf-rf* for text data with an example. Assume we have ocean and forest classes in a document classification setup. There are class-specific words such as *water* and *whale* for the ocean class and *tree* and *bird* for the forest class. These words are more important for the class prediction than stop words such as *a* and *the*. The word *whale* is more informative for ocean class prediction but has no significance in forest class prediction. *tf-rf* term assigns class specific weights to the words. For our example, we define two weights for the word *whale*, one for each class. The weight of the word *whale* calculated for the ocean class is greater than for the forest class. Therefore, it is more effective in predicting the ocean class than in predicting the forest class. Similarly, the weight of the word *tree* calculated for the forest class is greater than for the ocean class.

Inspired by these class-related words, we want to model some DNA mutations as disease-related mutations. We want to include them in the decision mechanism depending on their significance for the specific disease. If a mutation *m* is found significantly more often in patients with disease *d1* than in patients with disease *d2*, than we can conclude that it represents a higher indication for disease *d1*. With *tf-rf*, we want to assign weights for mutations proportional to their significance for the disease. This significance is presented in the relevance-frequency (*rf*) component. We expect to calculate higher *rf* values for cancer classes for the disease-related genes. For example, *rf* metrics calculated for GABRA3 are 1.184751598 for breast cancer and 1.025137749 for non-cancer patients. In [28], it is shown that high expression of GABRA3 is inversely correlated with breast cancer survival. Another example can be PLAC8. *rf* metrics calculated for PLAC8 are 1.181244553 for breast cancer and 1.027704319 for non-cancer patients. In [29], it is shown that higher PLAC8 expression was correlated with worse outcome and aggressive phenotype in breast cancer. Another example can be AKAP4. *rf* metrics calculated for AKAP4 are 1.171182982 for breast cancer and 1.035242675 for non-cancer patients. In [30], it is suggested that AKAP4 may be used as serum based diagnostic test for an early detection and diagnosis of breast cancer. From these examples, it is clearly seen that *tf-rf* metric assigns higher values to gene mutations which are related to specific cancer types. The difference between assigned values may be small, but with the interactions in the graph, they help to predict cancer cases with higher success rates.

The term-frequency (*tf*) in our domain is taken as the count of mutations of a gene in a patient. The relevance-frequency (*rf*) measures how important this gene mutation is for each class, in our case for cancer or non-cancer patients. The final *tf-rf* value is obtained by multiplying these two parameters as in Eq. 1. This reflects the degree of impact of gene mutations when considering the patient’s DNA mutations and also all DNA mutations for the patient’s class. Therefore, we can say that this metric summarizes both the personal and global mutation information for a patient.

For *bm25-tf-rf*, we have replaced the term *tf* in *tf-rf* with the term *tf* in the *BM25* formula [31] as in Eq. 2. The use of *bm25-tf* introduced a smoothing effect over *tf* with the parameter *k* and prevented the *tf* parameter from increasing too much depending on the number of mutations (Eq. 3). With *bm25-tf-rf*, the gene mutations are weighted according to their significance both for the patient (represented in the term $BM25\text {-}tf$) and for the patient’s class (represented in the term *rf*). *rf* term is calculated by comparing the gene mutation counts in classes with the terms *a* for the patient’s class and *b* for the other class, as shown in Eq. 4. The class information in the formula enables the class-related mutations to have higher feature values and therefore have a greater impact on the predictions. When a system is trained with this information, it can create an internal profile of the disease classes and make more accurate predictions based on this.

1 $$tf\!\text{-}rf = tf * rf$$

2 $$BM25\text{-}tf\!\text{-}rf = BM25\text{-}tf * rf$$

3 $$\begin{aligned} BM25\text {-}tf&= ((k+1)*tf)/(k+tf) \end{aligned}$$

4 $$\begin{aligned} rf&= log(2+a/max(1,b)) \end{aligned}$$

#### GNN model

After deciding on the heterogeneous graph structure and features, we need to select a graph network. There are different GNN types and implementations. The most commonly used graph network is GCN. But the GCN implementation of the PyG [32] library does not support graphs with edges connecting different node types. Also, we want to differentiate between mutation-based updates for a patient, as not every mutation affects disease development equally. Therefore, we chose GAT [33] as our graph network. With the attention mechanism, the network learns the edge weights during training and differentiates the effect of each node update by edges based on the input data.

#### Explainer model

Before explainer methods, classification algorithms used to be black box methods. Nowadays, interpretation libraries allow us to understand how these complex methods make decisions. In this study, we applied Captum [34] explainer to find the roots of the predictions of our heterogeneous graph based model. Our goal is to evaluate and compare the impact of gene mutations on the final class decisions for patients. We computed integrated gradients (IG) [35] on protein-patient edges for cancer patients. The edges with higher attribution values indicate that the gene producing the protein in the source of the edge has a greater impact on disease detection and development. To obtain attribution scores, we first transformed our heterogeneous model into a Captum heterogeneous model. Second, we converted our inputs from our heterogeneous graph into Captum inputs. Then, we computed the IG attribution algorithm for all protein-patient edges in our Captum inputs using our Captum heterogeneous model for cancer samples. We formulate our deep model as a function $\text {F}: R^n \rightarrow [0, 1]$. We have our feature vectors and a set of nodes and edges and our model aims to perform a binary classification. Let $x \in R^n$ be the input at hand, and $x' \in R^n$ be the baseline input. The integrated gradient along the i^th^ dimension for an input *x* and the baseline $x'$ is defined in Eq. 5.

5 $$\begin{aligned} \text {IG}_i(x) {:}{:}{=} (x_i - {x_i}') * \int _{\alpha =0}^1 \frac{\partial F(x' + \alpha (x-x'))}{\partial x_i} d\alpha \end{aligned}$$

### Implementation and experiment design

GNNMutation model is implemented in Python with the PyG library [32]. The best performing model for our heterogeneous graph is GAT with 2 levels. We tuned the network parameters with several tests. The reported results are with the best parameters, namely 128 for the number of hidden units, 8 for the number of GAT heads and 0.001 for the learning rate. We performed our tests with transductive learning. All tests were performed with 3 repeats of 10-fold cross-validation by keeping the rate of case–control samples equal in each fold. The average accuracy with standard deviation are used as a performance measure for comparisons. The best parameters were selected according to the accuracy on the validation set. Reported accuracies were calculated using the test set. We also report the sensitivity, specificity, Matthews correlation coefficient (MCC), the area under the curve (AUC), the area under the precision-recall curve (AUPRC) and f1-score metrics.

To determine the effect of the graph data model on classification performance, we selected algorithms without graph input for comparison. We applied logistic regression (LR) and multilayer perceptron (MLP) with 4 layers. We also applied Model-Half from our previous study [26]. To our knowledge, for VCF data and cancer classification, Model-Half provides the state-of-the-art results. We used the same patient feature vectors as input for these algorithms. For the graph-based comparison, we followed the general approach of most studies. We created a homogeneous graph for each patient which is the same PPI network used in the heterogeneous graph for GNNMutation. The nodes are the proteins and the edges are defined based on protein interactions. We assigned the patient features to the protein nodes in this graph such that each node is assigned the corresponding *bm25-tf-rf* value for that protein. This is a graph classification setup. For processing unit, the same GAT configuration is used as GNNMutation. We named this model as HomogeneousGNN.

## Results and discussion

### Comparison of different numbers of protein-patient edges

We defined our protein-patient edges based on the genes that have maximum feature values for each patient. We connected each patient to the *k* proteins that have the highest mutation scores in the patient’s feature vector. For the initial tests, we selected *k* as 100 genes for each patient. We repeated our tests with different number of genes. The classification test results with different number of protein-patient edges per patient are shown in Table 1. The validation set accuracy values are parallel to the test set results. For breast cancer, increasing the number of genes first leads to improved accuracy. However, when the number of edges exceeds a threshold, in our case 300 genes, the accuracy value starts to decrease. This may be due to the fact that, in graph networks, nodes can have similar values after certain runs when they are updated by similar edges. This similarity makes it difficult to differentiate between nodes and degrades performance. The highest accuracy for both breast and prostate cancer is achieved by selecting *k* as 300 genes.

**Table 1:** Classification test accuracy results with different number of protein-patient edges per patient (The highest scores are shown in bold, the standard deviation is given in paranthesis)

Edge	Breast	Prostate
Max 100	0.740 (0.008)	0.847 (0.003)
Max 200	0.824 (0.006)	0.800 (0.025)
Max 300	**0.903 (0.003)**	**0.871 (0.018)**
Max 400	0.873 (0.019)	0.841 (0.018)

### Comparison of different protein-patient edge strategies

The input for GNNMutation is a heterogeneous graph of protein and patient nodes. There are two types of edges in the input graph. The protein-protein edges were defined according to the PPI network. The protein-patient edges were defined based on the mutations in the genome of the patient. Each patient node is connected to the 300 proteins for which the corresponding genes of the patient have the highest (i.e., maximum) mutation scores. To validate our approach and demonstrate the impact of the mutation information, we also created a graph where each patient node is connected to 300 randomly selected protein nodes.

The classification test results for these two protein-patient edge strategies are shown in Table 2. The validation set accuracy values are parallel to the test set results. It can be seen from this table that maximum selection leads to higher accuracy for both cancer types. Random selection leads to poor results, demonstrating that incorporating PPI information with mutation information is essential and leads to a powerful approach for cancer detection.

**Table 2:** Classification test accuracy results with different protein-patient edge strategies. In the first strategy, each patient node is connected to the proteins for which the mutation scores in the patient feature vector are maximum. In the second strategy, each patient node is connected to random protein nodes. Thus, the first strategy incorporates mutation information, whereas the second strategy doesn’t (The highest scores are shown in bold, the standard deviation is given in paranthesis)

Edge	Breast	Prostate
Max 300	**0.903 (0.003)**	**0.871 (0.018)**
Random 300	0.495 (0.003)	0.495 (0.005)

### Comparison of algorithms

Classification test results are presented in Tables 3, 4, 5 and 6. We tested our data with three algorithms, LR, MLP and Model-Half, which don’t use a graph structure. LR leads to better accuracy compared to MLP for all four cancer types. LR also leads to higher f1-score compared to MLP in all our tests. Model-Half is more accurate compared to LR and MLP, which is consistent with our previous study [26]. For prostate cancer, LR and Model-Half performances are similar considering all of the performance metrics calculated in this study. When GNNMutation is considered, we find that the classification accuracy, MCC, AUC, AUPRC and f1-score values are significantly better than all three algorithms. For breast cancer, GNNMutation achieves an accuracy of $90.3\%$, for prostate cancer $87.1\%$, for lung cancer $81.3\%$ and for colon cancer $81.6\%$. The difference in all performance metrics indicates that GNNMutation is more stable and more accurate compared to non-graph based algorithms. The improved performance of GNNMutation is the result of the graph input model. By using the graph representation, the additional biological system information is appended to the features. And by using a GNN model, the messaging between the nodes enable the information transfer from proteins to patients. By modeling the GNN in multiple layers, not only mutation information but also the protein interactions are used to update the patient nodes.

We also compared our system with a homogeneous graph-based model. When we consider the classification results of HomogeneousGNN, we see that it cannot differentiate between case and control patients with the input parameters and the homogeneous graph structure. When we compare two graph-based methods, we see that GAT can learn from the data and is able to distinguish between different classes based on the definition of the heterogeneous graph structure.

If we look at Tables 1, 3 and 4 together, we can also see that even the lowest accuracy of the proposed system, GNNMutation, is better than all other methods tested in this study. If we look at the highest score of GNNMutation, it clearly outperforms the other methods and produces a significant leap in performance. When we consider sensitivity and specificity values, GNNMutation outperforms the other methods on these metrics as well. For breast cancer, the specificity of GNNMutation is higher. For prostate cancer, the sensitivity of GNNMutation is higher. But for each cancer type, the two metrics are close to each other. We can conclude that GNNMutation can model one class better, but overall it can distinguish between case and control patients with a higher classification performance compared to the other algorithms listed here.

**Table 3:** Classification test results for breast cancer (The highest scores are shown in bold, the standard deviation is given in paranthesis)

Model	Accuracy	Sensitivity	Specificity	MCC	AUC	AUPRC	F1-Score
LR	0.722 (0.000)	0.734 (0.000)	0.710 (0.000)	0.445 (0.000)	0.722 (0.000)	0.792 (0.000)	0.725 (0.000)
MLP	0.698 (0.006)	0.728 (0.050)	0.669 (0.063)	0.423 (0.017)	0.698 (0.006)	0.792 (0.001)	0.691 (0.022)
Model-Half	0.737 (0.004)	0.756 (0.012)	0.718 (0.017)	0.476 (0.008)	0.737 (0.004)	0.804 (0.003)	0.741 (0.003)
HomogeneousGNN	0.501 (0.000)	0.402 (0.000)	0.599 (0.000)	0.004 (0.000)	0.501 (0.000)	0.734 (0.000)	0.271 (0.000)
GNNMutation	**0.903 (0.003)**	**0.857 (0.002)**	**0.949 (0.003)**	**0.806 (0.003)**	**0.903 (0.003)**	**0.935 (0.002)**	**0.895 (0.001)**

**Table 4:** Classification test results for prostate cancer (The highest scores are shown in bold, the standard deviation is given in paranthesis)

Model	Accuracy	Sensitivity	Specificity	MCC	AUC	AUPRC	F1-Score
LR	0.803 (0.000)	0.793 (0.000)	0.813 (0.000)	0.609 (0.000)	0.803 (0.000)	0.854 (0.000)	0.801 (0.000)
MLP	0.746 (0.016)	0.764 (0.047)	0.729 (0.036)	0.517 (0.027)	0.746 (0.016)	0.833 (0.008)	0.733 (0.033)
Model-Half	0.805 (0.005)	0.808 (0.007)	0.801 (0.005)	0.611 (0.010)	0.805 (0.005)	0.854 (0.003)	0.805 (0.005)
HomogeneousGNN	0.499 (0.000)	0.600 (0.000)	0.399 (0.000)	0.007 (0.000)	0.499 (0.000)	0.700 (0.000)	0.400 (0.000)
GNNMutation	**0.871 (0.018)**	**0.874 (0.055)**	**0.868 (0.021)**	**0.757 (0.031)**	**0.871 (0.018)**	**0.910 (0.008)**	**0.867 (0.030)**

**Table 5:** Classification test results for lung cancer (The highest scores are shown in bold, the standard deviation is given in paranthesis)

Model	Accuracy	Sensitivity	Specificity	MCC	AUC	AUPRC	F1-Score
LR	0.722 (0.000)	0.716 (0.000)	0.728 (0.000)	0.446 (0.000)	0.722 (0.000)	0.792 (0.000)	0.719 (0.000)
MLP	0.661 (0.039)	0.665 (0.018)	0.657 (0.090)	0.352 (0.062)	0.661 (0.039)	0.775 (0.012)	0.625 (0.037)
Model-Half	0.732 (0.005)	0.736 (0.006)	0.728 (0.005)	0.465 (0.010)	0.732 (0.005)	0.799 (0.004)	0.733 (0.005)
HomogeneousGNN	0.500 (0.000)	0.600 (0.000)	0.400 (0.000)	0.002 (0.000)	0.500 (0.000)	0.750 (0.000)	0.400 (0.000)
GNNMutation	**0.813 (0.012)**	**0.853 (0.027)**	**0.773 (0.027)**	**0.645 (0.017)**	**0.813 (0.012)**	**0.869 (0.005)**	**0.820 (0.016)**

**Table 6:** Classification test results for colon cancer (The highest scores are shown in bold, the standard deviation is given in paranthesis)

Model	Accuracy	Sensitivity	Specificity	MCC	AUC	AUPRC	F1-Score
LR	0.716 (0.000)	0.723 (0.000)	0.709 (0.000)	0.433 (0.000)	0.716 (0.000)	0.787 (0.000)	0.718 (0.000)
MLP	0.675 (0.006)	0.674 (0.043)	0.676 (0.043)	0.382 (0.018)	0.675 (0.006)	0.777 (0.003)	0.644 (0.016)
Model-Half	0.736 (0.007)	0.755 (0.005)	0.718 (0.008)	0.474 (0.012)	0.736 (0.007)	0.804 (0.004)	0.741 (0.006)
HomogeneousGNN	0.500 (0.000)	0.600 (0.000)	0.400 (0.000)	0.002 (0.000)	0.500 (0.000)	0.750 (0.000)	0.400 (0.000)
GNNMutation	**0.816 (0.022)**	**0.826 (0.028)**	**0.806 (0.057)**	**0.656 (0.037)**	**0.816 (0.022)**	**0.876 (0.015)**	**0.816 (0.021)**

### Ablation study

GNNMutation has many components that work together to achieve high classification accuracy. In this ablation study, we subtracted one component of GNNMutation at a time to determine its contribution to the results. We decomposed four components of GNNMutation, namely the feature weighting technique, the GNN component, the graph structure, and the edge selection strategy. For four models in our ablation study, we excluded one component in each one. To exclude the GNN component, we tested our data with Model-Half by using the same *bm25-tf-rf* features. To exclude the heterogeneous graph structure, we tested our data with HomogeneousGNN using the same *bm25-tf-rf* features and the same PPI network. To rule out *bm25-tf-rf* feature weighting, we tested our data with a GNNMutation model using the same graph but normalized number of gene mutations as patient features. To exclude maximum edge selection strategy, we tested our data with a GNNMutation model using the same *bm25-tf-rf* features and the same PPI network but random protein-patient edges. The details of these models and the proposed GNNMutation model (last row) can be found in Table 7.

**Table 7:** Ablation study details for GNNMutation

Model	Feature weighting	GNN component	Graph structure	Edge selection
Model-Half	bm25-tf-rf	–	–	–
HomogeneousGNN	bm25-tf-rf	+	homogeneous	–
GNNMutation-rand	bm25-tf-rf	+	heterogeneous	Random
GNNMutation-nm	num. of mutations	+	heterogeneous	Max
GNNMutation	bm25-tf-rf	+	heterogeneous	Max

We tested these models with breast and prostate cancer datasets. The results for the ablation study models and the proposed GNNMutation model are presented in Table 8. As we can see from these results, the success of GNNMutation is dependent on all four components. If we exclude one of them, the performance decreases significantly. The graph structure and the edge selection strategy are the most effective components of our model. Changing these parts almost halves the performance. The *bm25-tf-rf* feature weighting technique can be mentioned as the second important component after these two components. If we keep the same feature weights but extract the graph structure at all, the performance is higher compared to other ablation results. This shows the representation power of the features. But there is still a significant difference with the proposed model. This difference is made by the heterogeneous graph representation. The patients are represented as separate nodes and this allows the system to reflect the effects of other biological concepts on the patient nodes and predict the cancer status as a result of to these updates.

**Table 8:** Ablation study results for GNNMutation (accuracy) (The highest scores are shown in bold, the standard deviation is given in paranthesis)

Model	Breast	Prostate
Model-Half (w/o GNN component)	0.737 (0.004)	0.802 (0.001)
HomogeneousGNN (w/o heterogeneous graph)	0.500 (0.000)	0.499 (0.000)
GNNMutation-rand (w/o max edge selection)	0.495 (0.003)	0.495 (0.005)
GNNMutation-nm (w/o bm25-tf-rf feature weights)	0.555 (0.010)	0.563 (0.011)
GNNMutation	**0.903 (0.003)**	**0.871 (0.018)**

### Discussion of explainer results

The input of the GNNMutation framework is a heterogeneous graph containing both patient and protein information. With the undirected protein-protein edges, the protein nodes update each other. With the directed protein-patient edges, the protein nodes update the patient nodes with mutation information and their interaction information. Graph-based frameworks make predictions that take these updates into account as well as the feature values. The explainer for the graph model extracts the rationale behind the framework’s decision mechanisms. We let the explainer evaluate the protein-patient nodes to see the effects of mutations and protein interactions on the predictions. We then select 20 genes with the highest scores for each cancer type. For visualization, we normalized the gene scores of the top 20 genes between 0 and 1.

**Fig. 3:**
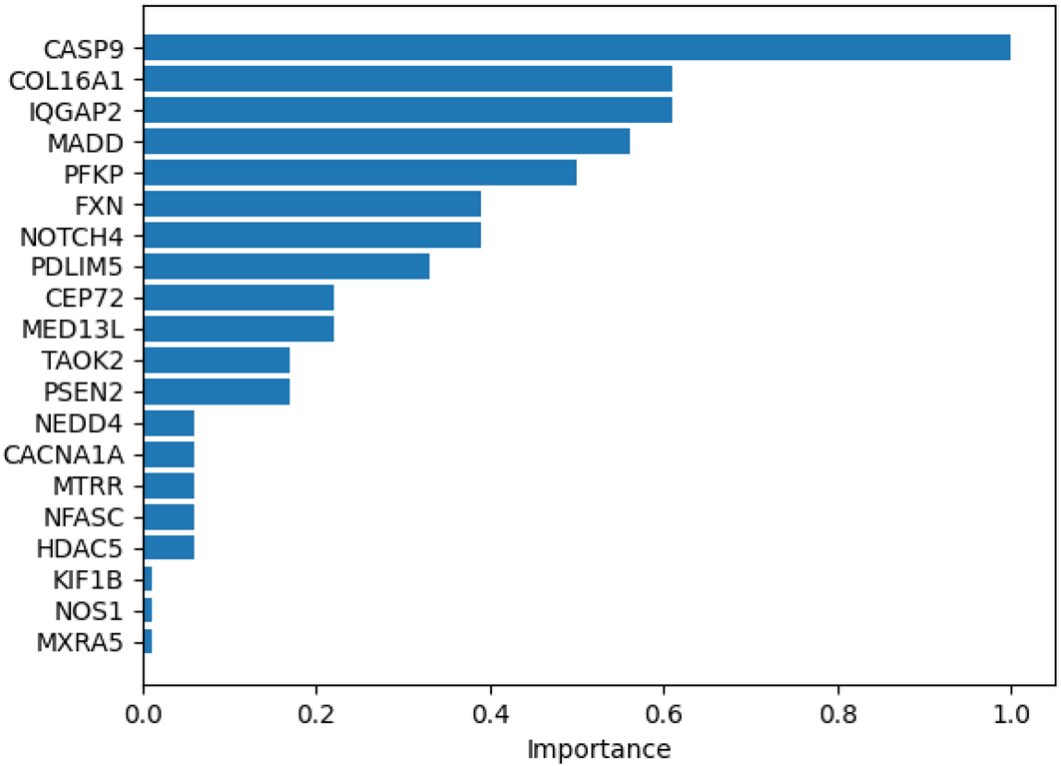
Explainer results for breast cancer

The most effective genes and their normalized scores for breast cancer are shown in Fig. 3. [36] suggests that CASP9 transcriptional regulation is an important factor in the development of breast cancer. [37] proposes CASP9 as a biomarker and a therapeutic target in inflammatory breast cancer. [38] investigates the role of IQGAP2 in tumor angiogenesis in breast cancer. [39] investigates the role of IQGAP2 as a tumor suppressor in breast cancer. [40] examines breast tissue microarrays and shows overexpression of MADD. [41] shows that the PFKP protein is highly expressed in triple-negative breast cancer (TNBC). [42] investigates that silencing NOTCH4 promotes tumorigenesis and inhibits metastasis in TNBC. [43] suggests that MED13L positively correlates with survival of patients with breast cancer. [44] finds that NEDD4 expression is a predictive factor for the response to hormone therapy in breast cancer patients. [45] indicates upregulated MXRA5 expression in breast cancer tissue.

**Fig. 4:**
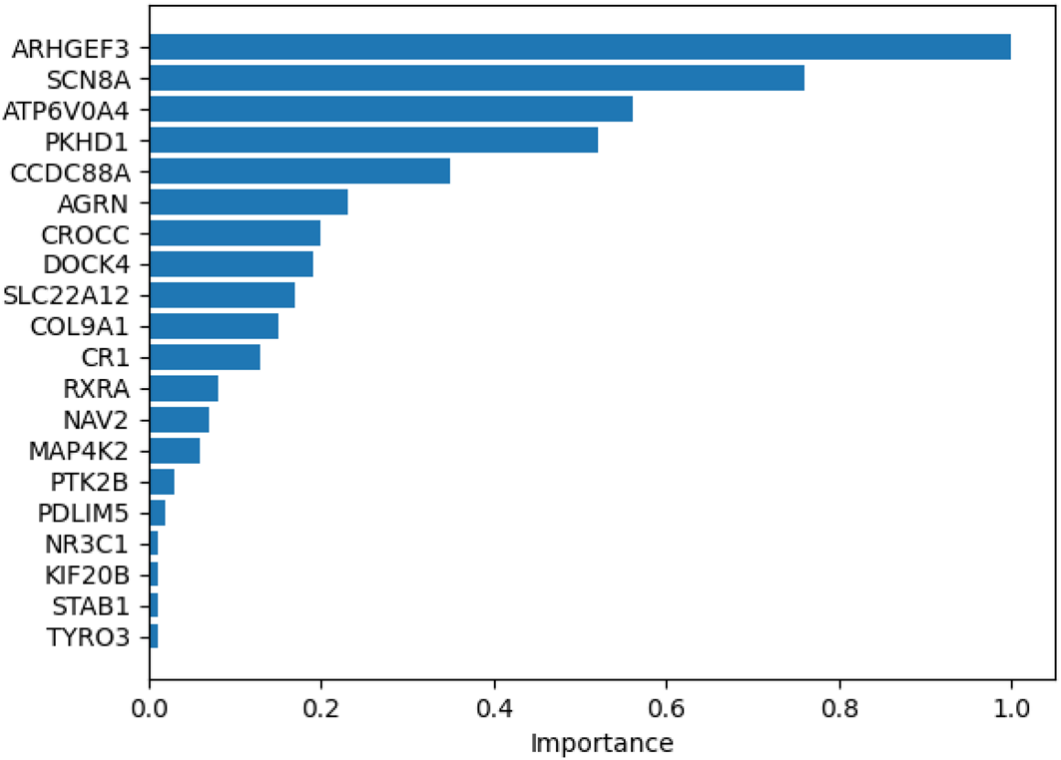
Explainer results for prostate cancer

The most effective genes and their normalized scores for prostate cancer are shown in Fig. 4. ARHGEF3 is shown to be an oncogene and is proposed as a novel biomarker for the prediction of invasive prostate cancer in [46]. [47] associates high and intermediate levels of CCDC88A with higher prostate cancer survival. [48] finds that overexpression of DOCK4 is observed in primary prostate tumors in the TCGA dataset. [49] finds that CR-1 mRNA and protein are upregulated in prostate cancer and suggests that CR-1 expression may be a novel biological target for personalized therapy. [50] identifies RXRA as a novel target in prostate cancer. PDLIM5 is reported to be abnormally enriched in prostate cancer tissues in [51]. According to [52], studies using various systems biology methods have highlighted NR3C1 as a potential gene in prostate cancer metastasis. SATB1 is shown to be overexpressed in metastatic prostate cancer in [53]. [54] finds that serum concentration of TYRO3 is significantly increased in men with localized or metastatic prostate cancer compared to men without prostate cancer.

We searched the literature if there exist research for top 20 of these proposed genes related with the corresponding cancer type by our explainer. We found that nearly half of them took part in previous studies. This shows that our framework emphasizes on proper genes for the selected cancer types. And also the rest of the genes that are proposed by the explainer can be utilized as novel target genes for new cancer studies.

### A real-life simulation experiment for GNNMutation

Since the patient features of GNNMutation are based on class information, we constructed a simulation study to demonstrate how it can be applied in a real-life scenario where the class information (cancer or no-cancer) of a patient is not known. In this case, we have to consider the patient as being in both classes and compare the results for the final prediction.

We combined our training and validation sets and used them as a large training set, which makes up $90\%$ of our dataset. The simulation test set consists of $10\%$ of our dataset. The class ratios are kept the same in all sets. For each test patient, we calculated two node feature vectors. We calculated one feature vector taking the patient as in case group and another feature vector taking the patient as in control group. For each test patient, we trained and tested GNNMutation twice with one of the feature vectors. We can illustrate this setup with an example. For breast cancer, we have a total of 2000 patients in our dataset; 1000 patients for the case group and 1000 patients for the control group. We selected 1800 of these as the training set and 200 as the test set for the simulation. For a case group patient, $P_c$, in the test set, we calculated a feature vector as the patient is in case group, $P_{c1}$, and also a feature vector as the patient is in control group, $P_{c2}$. We constructed two heterogeneous graphs: one with train set and $P_{c1}$ and one with train set and $P_{c2}$. We applied GNNMutation on these two graphs. We then calculated the sum of the prediction probabilities for the case and control classes from these two runs and then decided on the final class prediction based on the highest overall prediction probability. We repeated this for all 200 test patients. A single test run took about 18 minutes on an Intel i9 machine with 64GB ram, linux operating system and NVIDIA GeForce GTX 1080 Ti graphic card. Since we performed two test runs for each patient, a decision for a patient lasted 36 minutes after feature vectors are calculated. The required time for the simulation is constant for any patient and every patient can be tested in parallel independent from others. The prediction performance results of our simulation study are presented in Table 9. The simulation accuracy results are lower then test accuracy results as expected. However, the obtained performance is still promising and demonstrates the potential of the proposed method to be used as a non-invasive approach to aid cancer diagnosis and also predict causal genes.

**Table 9:** Classification results of GNNMutation for the real-life simulation experiment

Cancer type	Accuracy	Sensitivity	Specificity
Breast	0.800	0.880	0.720
Prostate	0.720	0.630	0.810

## Conclusion

DNA mutations can affect the structure and function of the corresponding proteins. These effects can change the behaviour of a cell from normal to cancerous [1]. In this study, we propose an explainable graph-based framework to detect the genomic roots of cancer. We selected the four most common cancers for our tests. We utilized gene mutations from whole exome sequencing data and enrich them with protein interaction data. We modeled our input graph to contain separate patient and protein nodes. With this heterogeneous graph structure, we can model the effects of gene mutations and protein-protein interactions on patient decisions by defining corresponding edges. We compared our framework, GNNMutation, with both graph-based and non-graph based algorithms. Our test results show that the updates in the heterogeneous graph structure lead to a significantly better discrimination between case and control patients. Furthermore, we employ an explainer module that examines GNNMutation and evaluates the genes according to their influence on cancer decision. Our literature search on these genes shows that many of them have already been studied and reported in association with the corresponding cancer types. Therefore, we suggest that the remaining genes in the explainer output list are candidate genes to be studied further for the selected cancer types. In addition, we created a simulation study to demonstrate how GNNMutation can be used in a real-life situation. GNNMutation has been trained and run on four cancer types but can be applied to any genomic disease.

## Data Availability

The source code of the proposed method, GNNMutation, is available at https://github.com/nozlemozcan/GNNMutation. The actual mutation data is taken from UK Biobank and the actual PPI network is taken from String DB. Sample preprocessed input files and their descriptions are also available at the repository.

## References

[1] The national cancer institute: (NCI). https://www.cancer.gov/

[2] Ramirez R, Chiu Y-C, Hererra A, Mostavi M, Ramirez J, Chen Y, Huang Y, Jin Y-F. Classification of cancer types using graph convolutional neural networks. In: Frontiers of Physics 2020. 10.3389/fphy.2020.00203 PMC7799442 33437754

[3] Schulte-Sasse R, Budach S, Hnisz D, Marsico A. Integration of multiomics data with graph convolutional networks to identify new cancer genes and their associated molecular mechanisms. Nat Mach Intell. 2021;3:513–26.

[4] Pfeifer B, Secic A, Saranti A, Holzinger A. Gnn-subnet: disease subnetwork detection with explainable graph neural networks. Bioinformatics. 2022;38:120–6. 10.1093/bioinformatics/btac478 36124793

[5] Zhang S, Xu J, Zhang T. Dgmp: identifying cancer driver genes by jointing dgcn and mlp from multi-omics genomic data. Genomics, Proteomics Bioinf. 2022;20:928–38. 10.1016/j.gpb.2022.11.004 PMC10025764 36464123

[6] Peng W, Wu R, Dai W, Yu N. Identifying cancer driver genes based on multi-view heterogeneous graph convolutional network and self-attention mechanism. BMC Bioinf. 2023;24(1):16. 10.1186/s12859-023-05140-3 PMC9838012 36639646

[7] Zhong Y, Peng Y, Lin Y, Chen D, Zhang H, Zheng W, Chen Y, Wu C. Modilm: towards better complex diseases classification using a novel multi-omics data integration learning model. BMC Med Inf Decis Making. 2023;23(1):82. 10.1186/s12911-023-02173-9 PMC10161645 37147619

[8] Li B, Nabavi S. A multimodal graph neural network framework for cancer molecular subtype classification. BMC Bioinf. 2024;25(1):27. 10.1186/s12859-023-05622-4 PMC10789042 38225583

[9] Chatzianastasis M, Vazirgiannis M, Zhang Z. Explainable multilayer graph neural network for cancer gene prediction. Bioinformatics. 2023;39(11):btad643. 37862225 10.1093/bioinformatics/btad643 PMC10636280

[10] Ren Y, Gao Y, Du W, Qiao W, Li W, Yang Q, Liang Y, Li G. Classifying breast cancer using multi-view graph neural network based on multi-omics data. Front Genet. 2024;15:1363896. 38444760 10.3389/fgene.2024.1363896 PMC10912483

[11] Rosa D, Pellicani A, Pio G, D’Elia D, Ceci M. Exploiting microrna expression data for the diagnosis of disease conditions and the discovery of novel biomarkers. In: International syposium on methodologies for intelligent systems, 2024;77–86

[12] Liu J, Xue X, Wen P, Song Q, Yao J, Ge S. Multi-fusion strategy network-guided cancer subtypes discovering based on multi-omics data. Front Genet. 2024;15:1466825. 39610828 10.3389/fgene.2024.1466825 PMC11602503

[13] Li X, Ma J, Leng L, Han M, Li M, He F, Zhu Y-p. Mogcn: a multi-omics integration method based on graph convolutional network for cancer subtype analysis. Front Genet. 2022;13: 806842. 35186034 10.3389/fgene.2022.806842 PMC8847688

[14] Xu Z, Liao H, Huang L, Chen Q, Lan W, Li S. Ibpgnet: lung adenocarcinoma recurrence prediction based on neural network interpretability. Brief Bioinf. 2024;25(3):080. 10.1093/bib/bbae080 PMC10982951 38557672

[15] Nunes L, Li F, Wu M, Luo T, Hammarstrom K, Torell E, Ljuslinder I, Mezheyeuski A, Edqvist P-HD, Lofgren-Burstrom A, Zingmark C, Edin S, Larsson C, Mathot L, Osterman E, Osterlund E, Ljungstrom V, Neves I, Yacoub N, Gudnadottir U, Birgisson H, Enblad M, Ponten F, Palmqvist R, Xu X, Uhlen M, Wu K, Glimelius B, Lin C, Sjoblom T. Prognostic genome and transcriptome signatures in colorectal cancers. Nature. 2024;633:137–46. 39112715 10.1038/s41586-024-07769-3 PMC11374687

[16] Cornish AJ, Gruber AJ, Kinnersley B, Chubb D, Frangou A, Caravagna G, Noyvert B, Lakatos E, Wood HM, Thorn S, Culliford R, Arnedo-Pac C, Househam J, Cross W, Sud A, Law PJ, Leathlobhair MN, Hawari A, Woolley C, Sherwood K, Feeley N, Gül G, Fernandez-Tajes J, Zapata L, Alexandrov LB, Murugaesu N, Sosinsky A, Mitchell J, López-Bigas N, Quirke P, Church DN, Tomlinson IPM, Sottoriva A, Graham TA, Wedge DC, Houlston RS. The genomic landscape of 2,023 colorectal cancers. Nature. 2024;633:127–36. 39112709 10.1038/s41586-024-07747-9 PMC11374690

[17] Braun P, Gingras A-C. History of protein-protein interactions: From egg?white to complex networks. PROTEOMICS. 2012;12. 10.1002/pmic.201100563 22711592

[18] Tanvir RB, Islam MM, Sobhan M, Luo D, Mondal AM. Mogat: a multi-omics integration framework using graph attention networks for cancer subtype prediction. Int J Mol Sci. 2024;25(5):2788. 38474033 10.3390/ijms25052788 PMC10932030

[19] Zhang G, Ma C, Yan C, Luo H, Wang J, Liang W, Luo J. Msfn: a multi-omics stacked fusion network for breast cancer survival prediction. Front Genet. 2024;15:1378809. 39161422 10.3389/fgene.2024.1378809 PMC11331006

[20] Elbashir MK, Almotilag A, Mahmood MA, Mohammed M. Enhancing non-small cell lung cancer survival prediction through multi-omics integration using graph attention network. Diagnostics. 2024;14(19):2178. 39410583 10.3390/diagnostics14192178 PMC11475495

[21] UKBiobank: (UKBiobank). https://www.ukbiobank.ac.uk/

[22] American Cancer Society: (ACS). https://www.cancer.org/

[23] VCF Specification: (2022). https://samtools.github.io/hts-specs/VCFv4.2.pdf

[24] Hallmark: (Hallmark). https://www.gsea-msigdb.org/gsea/msigdb/collections.jsp/

[25] StringDB: (StringDB). https://string-db.org/

[26] Ozcan Simsek NO, Ozgur A, Gurgen F. Statistical representation models for mutation information within genomic data. BMC Bioinf. 2019;20:324. 10.1186/s12859-019-2868-4 PMC6567431 31195961

[27] Lan M, Tan CL, Su J, Lu Y. Supervised and traditional term weighting methods for automatic text categorization. IEEE Trans Pattern Anal Mach Intell. 2009;31:721–35. 19229086 10.1109/TPAMI.2008.110

[28] Gumireddy K, Li A, Kossenkov AV, Sakurai M, Yan J, Li Y, Xu H, Wang J, Zhang PJL, Zhang L, Showe LC, Nishikura K, Huang Q. The mrna-edited form of gabra3 suppresses gabra3-mediated akt activation and breast cancer metastasis. Nat Commun. 2016;7(1):10715. 26869349 10.1038/ncomms10715 PMC4754346

[29] Chen Y, Jia Y, Mao M, Gu Y, Xu C, Yang J, Hu W-X, Shen J, Hu D, Chen C, Li Z, Chen L, Ruan J, Shen P, Zhou J, Wei Q, Wang L. Plac8 promotes adriamycin resistance via blocking autophagy in breast cancer. J Cell Mol Med. 2021;25:6948–62. 34117724 10.1111/jcmm.16706 PMC8278087

[30] Saini S, Jagadish N, Gupta A, Bhatnagar A, Suri A. A novel cancer testis antigen, a-kinase anchor protein 4 (akap4) is a potential biomarker for breast cancer. PLoS ONE. 2013;8(2): e57095. 23451156 10.1371/journal.pone.0057095 PMC3579772

[31] Jones KS, Walker S, Robertson SE. A probabilistic model of information retrieval: development and comparative experiments - part 1. Inf Process Manag. 2000;36:779–808.

[32] PyG: (PyG). https://pyg.org/

[33] Velickovic P, Cucurull G, Casanova A, Romero A, Lio’ P, Bengio Y. Graph attention networks. 2017. arxiv: abs/1710.10903

[34] Captum: (Captum). https://captum.ai/

[35] Sundararajan M, Taly A, Yan Q. Axiomatic attribution for deep networks. In: international conference on machine learning 2017.

[36] Theodoropoulos GE, Michalopoulos NV, Pantou MP, Kontogianni P, Gazouli M, Karantanos T, Lymperi M, Zografos GC. Caspase 9 promoter polymorphisms confer increased susceptibility to breast cancer. Cancer Genet. 2012;205(10):508–12. 22981751 10.1016/j.cancergen.2012.08.001

[37] Zhang M, Wu K, Wang M, Bai F, Chen H. 2021 Casp9 as a prognostic biomarker and promising drug target plays a pivotal role in inflammatory breast cancer. Int J Anal Chem. 2022;1:1043445. 10.1155/2022/1043445 PMC9527435 36199443

[38] Kumar D, Patel SA, Khan R, Chawla S, Mohapatra N, Dixit M. Iq motif-containing gtpase-activating protein 2 inhibits breast cancer angiogenesis by suppressing vegfr2–akt signaling. Mol Cancer Res. 2021;20:77–91. 34615693 10.1158/1541-7786.MCR-20-1044

[39] Kumar D, Patel SA, Hassan MK, Mohapatra N, Pattanaik N, Dixit M. Reduced iqgap2 expression promotes emt and inhibits apoptosis by modulating the mek-erk and p38 signaling in breast cancer irrespective of er status. Cell Death Dis. 2021;12(4):389. 33846302 10.1038/s41419-021-03673-0 PMC8041781

[40] Turner A, Li L-C, Pilli T, Qian L, Wiley EL, Setty S, Christov KT, Ganesh L, Maker AV, Li P, Kanteti P, Gupta TKD, Prabhakar BS. Madd knock-down enhances doxorubicin and trail induced apoptosis in breast cancer cells. PLoS ONE. 2013;8(2): e56817. 23457619 10.1371/journal.pone.0056817 PMC3574069

[41] Peng Z-M, Han X, Wang T, Li J-J, Yang C-X, Tou F-F, Zhang Z. Pfkp deubiquitination and stabilization by usp5 activate aerobic glycolysis to promote triple-negative breast cancer progression. Breast Cancer Res. 2024;26(1):10. 38217030 10.1186/s13058-024-01767-z PMC10787506

[42] Tian Y, Zhang P, Mou Y, Yang W, Zhang J, Li Q, Dou X. Silencing notch4 promotes tumorigenesis and inhibits metastasis of triple-negative breast cancer via nanog and cdc42. Cell Death Discov. 2023;9(1):148. 37149651 10.1038/s41420-023-01450-w PMC10164131

[43] Eini M, Parsi S, Barati M, Bahramali G, Zarei MA, Kiani J, Azarnezhad A, Hosseini A. Bioinformatic investigation of micro rna-802 target genes, protein networks, and its potential prognostic value in breast cancer. Avicenna J Med Biotechnol. 2022;14:154–64. 35633990 10.18502/ajmb.v14i2.8882 PMC9077654

[44] Wan L, Liu T, Hong Z, Pan Y, Sizemore ST, Zhang J, Ma Z. Nedd4 expression is associated with breast cancer progression and is predictive of a poor prognosis. Breast Cancer Res: BCR. 2019;21:1–16. 31856858 10.1186/s13058-019-1236-7 PMC6923956

[45] Deng H, Wen C, Jiang S, Yu Y, Zhao J, Zhang B. Single-cell analysis reveals one cancer-associated fibroblasts subtype linked to metastasis in breast cancer: Mxra5 as a potential novel marker for prognosis. Am J Cancer Res. 2024;14(2):526–44. 38455411 10.62347/DDII2115 PMC10915337

[46] Liu Q-W, Li J-Y, Zhang X, Liu Y, Liu Q-Y, Xiao L, Zhang W-J, Wu H-Y, Deng K-Y, Xin H-B. Human amniotic mesenchymal stem cells inhibit hepatocellular carcinoma in tumour?bearing mice. J Cell Mol Med. 2020;24:10525–41. 32798252 10.1111/jcmm.15668 PMC7521292

[47] Suetens A, Moreels M, Quintens R, Chiriotti S, Tabury K, Michaux A, Grégoire V, Baatout S. Carbon ion irradiation of the human prostate cancer cell line pc3: a whole genome microarray study. Int J Oncol. 2014;44:1056–72. 24504141 10.3892/ijo.2014.2287 PMC3977812

[48] Mei Y, Li K, Zhang Z, Li M, Yang H, Wang H, Huang X, Li X, Shi S, Yang H. mir-33b-3p acts as a tumor suppressor by targeting dock4 in prostate cancer. Front Oncol. 2021;11: 740452. 34804930 10.3389/fonc.2021.740452 PMC8595470

[49] Liu Y, Wang J, Yang T, Liu R, Xu Y. Overexpression levels of cripto-1 predict poor prognosis in patients with prostate cancer following radical prostatectomy. Oncol Lett. 2019;18(3):2584–91. 31452743 10.3892/ol.2019.10555 PMC6676627

[50] Ray J, Haughey C, Hoey C, Jeon J, Murphy RG, Dura-Perez L, Mccabe N, Downes MR, Jain S, Boutros PC, Mills IG, Liu SK. mir-191 promotes radiation resistance of prostate cancer through interaction with rxra. Cancer Lett. 2019;473:107–17. 31874245 10.1016/j.canlet.2019.12.025

[51] Liu X, Chen L, Huang H, Lv J, Chen M, Qu F-J, Pan X-W, Li L, Yin L, Cui X-G, Gao Y, Xu D. High expression of pdlim5 facilitates cell tumorigenesis and migration by maintaining ampk activation in prostate cancer. Oncotarget. 2017;8:98117–34. 29228678 10.18632/oncotarget.20981 PMC5716718

[52] Shinawi T, Nasser KK, Moradi FA, Mujalli A, Albaqami WF, Almukadi HS, Elango R, Shaik NA, Banaganapalli B. A comparative mrna- and mirna transcriptomics reveals novel molecular signatures associated with metastatic prostate cancers. Front Genet. 2022;13:1066118. 36468011 10.3389/fgene.2022.1066118 PMC9708707

[53] Mao L, Yang C-H, Wang J-Q, Li W, Wen R, Chen J-C, Zheng J. Satb1 is overexpressed in metastatic prostate cancer and promotes prostate cancer cell growth and invasion. J Transl Med. 2013;11:111–111. 23642278 10.1186/1479-5876-11-111 PMC3651708

[54] Jansen FH, Rijswijk AL, Teubel WJ, Weerden WM, Reneman S, Bemd G-J, Roobol MJ, Bangma CH, Staal FJT, Jenster GW. Profiling of antibody production against xenograft-released proteins by protein microarrays discovers prostate cancer markers. J Proteome Res. 2012;11(2):728–35. 22136385 10.1021/pr2006473

